# Supplementary material for: Resolving the full spectrum of human genome variation using Linked-Reads
Source: Genome Res. 2019 Apr;29(4):635–45. doi: 10.1101/gr.234443.118 (PMC6442396; doi:10.1101/gr.234443.118)
Supplement: Supplemental Material [file supp_gr.234443.118_Supplemental_longranger-2.2.zip › longranger-2.2.tar.bz2!/lr2.2-public/lib/stan/cmdstan/stan/doc/api/html/classstan_1_1io_1_1mcmc__writer.html]

Stan: stan::io::mcmc\_writer< M, SampleRecorder, DiagnosticRecorder, MessageRecorder > Class Template Reference


|  |  |
| --- | --- |
|  | Stan  2.5.0  probability, sampling & optimization |


- Main Page
- Related Pages
- Namespaces
- Classes
- Files

- Class List
- Class Index
- Class Hierarchy
- Class Members

All Classes Namespaces Files Functions Variables Typedefs Enumerations Enumerator Friends Macros Pages

- stan
- io
- mcmc\_writer

Public Member Functions |
List of all members

stan::io::mcmc\_writer< M, SampleRecorder, DiagnosticRecorder, MessageRecorder > Class Template Reference

mcmc\_writer writes out headers and samples
More...

`#include <mcmc_writer.hpp>`

|  |  |
| --- | --- |
| Public Member Functions | |
|  | mcmc\_writer (SampleRecorder &sample\_recorder, DiagnosticRecorder &diagnostic\_recorder, MessageRecorder &message\_recorder, std::ostream \*msg\_stream=0) |
|  | Constructor. More... |
|  | |
| void | write\_sample\_names (stan::mcmc::sample &sample, stan::mcmc::base\_mcmc \*sampler, M &model) |
|  | Outputs parameter string names. More... |
|  | |
| template<class RNG > | |
| void | write\_sample\_params (RNG &rng, stan::mcmc::sample &sample, stan::mcmc::base\_mcmc &sampler, M &model) |
|  | Outputs samples. More... |
|  | |
| template<class Recorder > | |
| void | write\_adapt\_finish (stan::mcmc::base\_mcmc \*sampler, Recorder &recorder) |
|  | Internal method. More... |
|  | |
| void | write\_adapt\_finish (stan::mcmc::base\_mcmc \*sampler) |
|  | Prints additional info to the streams. More... |
|  | |
| void | write\_diagnostic\_names (stan::mcmc::sample sample, stan::mcmc::base\_mcmc \*sampler, M &model) |
|  | Print diagnostic names. More... |
|  | |
| void | write\_diagnostic\_params (stan::mcmc::sample &sample, stan::mcmc::base\_mcmc \*sampler) |
|  | Print diagnostic params to the diagnostic stream. More... |
|  | |
| template<class Recorder > | |
| void | write\_timing (double warmDeltaT, double sampleDeltaT, Recorder &recorder) |
|  | Internal method. More... |
|  | |
| void | write\_timing (double warmDeltaT, double sampleDeltaT) |
|  | Print timing information to all streams. More... |
|  | |

## Detailed Description

### template<class M, class SampleRecorder, class DiagnosticRecorder, class MessageRecorder> class stan::io::mcmc\_writer< M, SampleRecorder, DiagnosticRecorder, MessageRecorder >

mcmc\_writer writes out headers and samples

Template Parameters
:   |  |  |
    | --- | --- |
    | M | Model class |
    | SampleRecorder | Class for recording samples |
    | DiagnosticRecorder | Class for diagnostic samples |

Definition at line 29 of file mcmc\_writer.hpp.

## Constructor & Destructor Documentation

template<class M , class SampleRecorder , class DiagnosticRecorder , class MessageRecorder >

|  |  |  |  |
| --- | --- | --- | --- |
| stan::io::mcmc\_writer< M, SampleRecorder, DiagnosticRecorder, MessageRecorder >::mcmc\_writer | ( | SampleRecorder & | *sample\_recorder*, |
|  |  | DiagnosticRecorder & | *diagnostic\_recorder*, |
|  |  | MessageRecorder & | *message\_recorder*, |
|  |  | std::ostream \* | *msg\_stream* = `0` |
|  | ) |  |  |

inline

Constructor.

Parameters
:   |  |  |
    | --- | --- |
    | sample\_stream | samples are "written" to this stream (can abstract this?) |
    | diagnostic\_stream | diagnostic information is "written" to this stream |
    | msg\_stream | messages are output to this stream |

Precondition
:   arguments == 0 if and only if they are not meant to be used

Postcondition
:   none

Side effects: streams are stored in this object

Definition at line 51 of file mcmc\_writer.hpp.

## Member Function Documentation

template<class M , class SampleRecorder , class DiagnosticRecorder , class MessageRecorder >

template<class Recorder >

|  |  |  |  |
| --- | --- | --- | --- |
| void stan::io::mcmc\_writer< M, SampleRecorder, DiagnosticRecorder, MessageRecorder >::write\_adapt\_finish | ( | stan::mcmc::base\_mcmc \* | *sampler*, |
|  |  | Recorder & | *recorder* |
|  | ) |  |  |

inline

Internal method.

Prints additional sampler info to the stream.

Parameters
:   |  |  |
    | --- | --- |
    | sampler | sampler |
    | stream | stream to output stuff to |

Definition at line 139 of file mcmc\_writer.hpp.

template<class M , class SampleRecorder , class DiagnosticRecorder , class MessageRecorder >

|  |  |  |  |  |  |
| --- | --- | --- | --- | --- | --- |
| void stan::io::mcmc\_writer< M, SampleRecorder, DiagnosticRecorder, MessageRecorder >::write\_adapt\_finish | ( | stan::mcmc::base\_mcmc \* | *sampler* | ) |  |

inline

Prints additional info to the streams.

Prints to both the sample stream and the diagnostic stream

Parameters
:   |  |  |
    | --- | --- |
    | sampler | sampler |

Definition at line 157 of file mcmc\_writer.hpp.

template<class M , class SampleRecorder , class DiagnosticRecorder , class MessageRecorder >

|  |  |  |  |
| --- | --- | --- | --- |
| void stan::io::mcmc\_writer< M, SampleRecorder, DiagnosticRecorder, MessageRecorder >::write\_diagnostic\_names | ( | stan::mcmc::sample | *sample*, |
|  |  | stan::mcmc::base\_mcmc \* | *sampler*, |
|  |  | M & | *model* |
|  | ) |  |  |

inline

Print diagnostic names.

Parameters
:   |  |  |
    | --- | --- |
    | sample | unconstrained sample |
    | sampler | sampler |
    | model | model |

Precondition
:   sample, sampler, and model are consistent.

Postcondition
:   none

Side effects: diagnostic\_stream\_ is appended with comma
:   separated names with newline at the end

Definition at line 175 of file mcmc\_writer.hpp.

template<class M , class SampleRecorder , class DiagnosticRecorder , class MessageRecorder >

|  |  |  |  |
| --- | --- | --- | --- |
| void stan::io::mcmc\_writer< M, SampleRecorder, DiagnosticRecorder, MessageRecorder >::write\_diagnostic\_params | ( | stan::mcmc::sample & | *sample*, |
|  |  | stan::mcmc::base\_mcmc \* | *sampler* |
|  | ) |  |  |

inline

Print diagnostic params to the diagnostic stream.

Parameters
:   |  |  |
    | --- | --- |
    | sample | unconstrained sample |
    | sampler | sampler |

Precondition
:   sample and sampler are consistent

Postcondition
:   none.

Side effects: diagnostic\_stream\_ is appended with csv values of the
:   sample's get\_sample\_params(), the sampler's get\_sampler\_params(), and get\_sampler\_diagnostics()

Definition at line 203 of file mcmc\_writer.hpp.

template<class M , class SampleRecorder , class DiagnosticRecorder , class MessageRecorder >

|  |  |  |  |
| --- | --- | --- | --- |
| void stan::io::mcmc\_writer< M, SampleRecorder, DiagnosticRecorder, MessageRecorder >::write\_sample\_names | ( | stan::mcmc::sample & | *sample*, |
|  |  | stan::mcmc::base\_mcmc \* | *sampler*, |
|  |  | M & | *model* |
|  | ) |  |  |

inline

Outputs parameter string names.

First outputs the names stored in the sample object (stan::mcmc::sample), then uses the sampler provided to output sampler specific names, then adds the model constrained parameter names.

The names are written to the sample\_stream as comma separated values with a newline at the end.

Parameters
:   |  |  |
    | --- | --- |
    | sample | a sample (unconstrained) that works with the model |
    | sampler | a stan::mcmc::base\_mcmc object |
    | model | the model |

Precondition
:   none

Postcondition
:   none

Side effects: sample\_stream\_ is written to with comma separated values
:   with a newline at the end

Definition at line 79 of file mcmc\_writer.hpp.

template<class M , class SampleRecorder , class DiagnosticRecorder , class MessageRecorder >

template<class RNG >

|  |  |  |  |
| --- | --- | --- | --- |
| void stan::io::mcmc\_writer< M, SampleRecorder, DiagnosticRecorder, MessageRecorder >::write\_sample\_params | ( | RNG & | *rng*, |
|  |  | stan::mcmc::sample & | *sample*, |
|  |  | stan::mcmc::base\_mcmc & | *sampler*, |
|  |  | M & | *model* |
|  | ) |  |  |

inline

Outputs samples.

First outputs the values of the sample params from a stan::mcmc::sample, then outputs the values of the sampler params from a stan::mcmc::base\_mcmc, then finally outputs the values of the model.

The samples are written to the sample\_stream as comma separated values with a newline at the end.

Parameters
:   |  |  |
    | --- | --- |
    | rng | random number generator (used by model.write\_array()) |
    | sample | the sample in constrained space |
    | sampler | the sampler |
    | model | the model |

Definition at line 107 of file mcmc\_writer.hpp.

template<class M , class SampleRecorder , class DiagnosticRecorder , class MessageRecorder >

template<class Recorder >

|  |  |  |  |
| --- | --- | --- | --- |
| void stan::io::mcmc\_writer< M, SampleRecorder, DiagnosticRecorder, MessageRecorder >::write\_timing | ( | double | *warmDeltaT*, |
|  |  | double | *sampleDeltaT*, |
|  |  | Recorder & | *recorder* |
|  | ) |  |  |

inline

Internal method.

Prints timing information

Parameters
:   |  |  |
    | --- | --- |
    | warmDeltaT | warmup time in seconds |
    | sampleDeltaT | sample time in seconds |
    | stream | output stream |
    | prefix | prepend each line with the prefix; default is "" |

Precondition
:   none

Postcondition
:   none

Side effects: stream is updated with information about timing

Definition at line 231 of file mcmc\_writer.hpp.

template<class M , class SampleRecorder , class DiagnosticRecorder , class MessageRecorder >

|  |  |  |  |
| --- | --- | --- | --- |
| void stan::io::mcmc\_writer< M, SampleRecorder, DiagnosticRecorder, MessageRecorder >::write\_timing | ( | double | *warmDeltaT*, |
|  |  | double | *sampleDeltaT* |
|  | ) |  |  |

inline

Print timing information to all streams.

Parameters
:   |  |  |
    | --- | --- |
    | warmDeltaT | warmup time (sec) |
    | sampleDeltaT | sample time (sec) |

Definition at line 267 of file mcmc\_writer.hpp.

---

The documentation for this class was generated from the following file:

- src/stan/io/mcmc\_writer.hpp


---

[ Stan Home Page ]

*© 2011–2014,
Stan Development Team.*
